# Green Synthesis of Copper Nanoparticles Using Cotton

**DOI:** 10.3390/polym13121906

**Published:** 2021-06-08

**Authors:** Marissa Pérez-Alvarez, Gregorio Cadenas-Pliego, Odilia Pérez-Camacho, Víctor E. Comparán-Padilla, Christian J. Cabello-Alvarado, Esmeralda Saucedo-Salazar

**Affiliations:** 1 Centro de Investigación en Química Aplicada, Departamento de Síntesis de Polímeros, Saltillo 25294, Coahuila, Mexico; odilia.perez@ciqa.edu.mx (O.P.-C.); victor.comparan@ciqa.edu.mx (V.E.C.-P.); christian.cabello@ciqa.edu.mx (C.J.C.-A.); esmeralda.saucedo@ciqa.edu.mx (E.S.-S.); 2 CONACYT-Centro de Investigación y de Innovación del Estado de Tlaxcala, Tlaxcala 90000, Mexico

**Keywords:** Cu nanoparticles, green synthesis, cotton, chemical reduction

## Abstract

Copper nanoparticles (CuNP) were obtained by a green synthesis method using cotton textile fibers and water as solvent, avoiding the use of toxic reducing agents. The new synthesis method is environmentally friendly, inexpensive, and can be implemented on a larger scale. This method showed the cellulose capacity as a reducing and stabilizing agent for synthetizing Cellulose–Copper nanoparticles (CCuNP). Nanocomposites based on CCuNP were characterized by XRD, TGA, FTIR and DSC. Functional groups present in the CCuNP were identified by FTIR analysis, and XRD patterns disclosed that nanoparticles correspond to pure metallic Cu°, and their sizes are at a range of 13–35 nm. Results demonstrated that CuNPs produced by the new method were homogeneously distributed on the entire surface of the textile fiber, obtaining CCuNP nanocomposites with different copper wt%. Thus, CuNPs obtained by this method are very stable to oxidation and can be stored for months. Characterization studies disclose that the cellulose crystallinity index (CI) is modified in relation to the reaction conditions, and its chemical structure is destroyed when nanocomposites with high copper contents are synthesized. The formation of CuO nanoparticles was confirmed as a by-product, through UV spectroscopy, in the absorbance range of 300–350 nm.

## 1. Introduction

Nanotechnology is gaining strength in all areas of science and technology, because nanometric materials and nanoparticles have peculiar and unique properties that allow their application in different fields of sciences, such as agriculture, medicine aeronautics, cosmetics, electrics, biochemistry, mechanics, energy, etc. [1,2,3,4,5].

More recently, nanoparticles have raised great interest within the medical field due to their antiviral and antimicrobial properties, which make them an excellent focus of attention for conducting research to help combat infectious diseases, as they have shown great potential to solve different challenges [1,6,7]. There are a wide variety of synthesis methods or routes to obtain nanoparticles, known as chemical reduction, sol-gel, chemical vapor deposition (CVD), biochemical synthesis, green chemical synthesis, and sputtering synthesis, among others [8,9,10,11,12,13,14,15]. The particle size, morphology, and structure, as well as the antimicrobial activity, are influenced by the method by which the nanoparticle was synthesized. However, factors such as agglomeration and rapid oxidation have made it a difficult research area. On one hand, though extensive research has been done in noble metals such as silver (Ag) and gold (Au), their studies are limited due to their high costs. On the other hand, copper (Cu) is a key trace element for humans, plants, and animals [16,17], it is also abundant, much less expensive than other noble metals and has been recognized as antimicrobial materials by the US Environmental Protection Agency (EPA) [18]. The fame of copper increased recently with the COVID-19 pandemic, by reporting that SARS-CoV-2 on copper surfaces decomposes faster (4 h) compared to other materials such as plastic and stainless steel [19].

CuNPs synthesis can be easily developed by different methods; all of them have advantages and disadvantages. In the last years, the methods friendly with ambiance have gained a lot of interest. In the following, some reports about copper nanoparticles are described in a general manner.

Huang et al. describe the synthesis of copper nanoparticles with spherical shape (approximately size of 20 nm) encapsulated by multilayer graphene through chemical vapor deposition with copper acetylacetonate (II) powder as precursor [20]. Lee et al. [21] inform that CuNPs obtained by polyol method exhibited diameters of 61 ± 12 nm with spherical-shape, using polyvinylpyrrolidone (PVP) and L-ascorbic acid as protective agents (8 nm thick PVP coating), where the reducing agent was sodium acid hypophosphite. Thereafter, it has also been reported that the CuNPs synthesis uses natural polymers as chitosan; where, according to Sani-Usman et al.’s [22] investigation, the obtained CuNPs present spherical shapes and sizes in the range of 5–300 nm, depending on the chitosan concentrations. For this synthesis, a combination of chitosan and ascorbic acid, with NaOH and hydrazine was utilized.

Over the past few decades, green synthesis of nanomaterials based on plants or organic materials is an emerging field in nanoscience, with emphasis on avoidance of the use of toxic chemicals that support the development of ecofriendly techniques. There are many investigations that describe the metallic NP synthesis employing plant extracts or microbes (bacteria, fungi), where the importance in this process is due to the active biological component itself acting as a capping and reducing agent, decreasing the overall cost of the synthesis process. In this sense, sometimes external experimental conditions such as high energy and high pressure are not required, which leads to energy saving and eco-friendly processes. For example, an investigation reported by Sharmila et al. [14], where copper oxide (II) (CuO NPs) synthesis was performed with Cassia Ariculata L. leaves, and the CuO NPs had a size range between 30–35 nm with a spherical shape. One disadvantage of this method is that the extract needs to be stored at 4 °C for further use, and the mix of an extract with the copper sulfate should be incubated for 4 days at 37 °C [14]. When the Cu NPs were synthesized using an extract of Tinospora Cardifolia leaves as a capping and reducing agent, Cu NPs with size in a range of 63.3–143.0 nm and spherical shape were obtained. In addition, the XRD pattern points out the coexistence of two crystalline phases such as metallic copper and copper oxide nanoparticles, which is a drawback of this method [23]. On the other hand, Gupta et al., synthesized Cu nanoparticles using a *Fusarium oxysporum*, an anamorphic and ubiquitous fungus. The obtained NPs had spherical shapes or elongated spheres with a size of 13 nm; however, in this research, the drawback is the inoculation of fungus for being used as a bio-reducing, stabilizing and capping agent in CuNPs synthesis, since they require two days of shaking at 150 rpm [15]. Regarding other methods, Arunkumar et al., performed investigations by sol-gel technique, for obtaining CuO NPs from Lantana camara leaf extracts using CuCl_2_ ·2H_2_O as the salt precursor, NaOH, and sterile distilled water for treating the plant. They obtained CuO NPs with sizes among 17–21 nm and seed-spheroid in shape and agglomerated structures; one important drawback of this method was the need for titration with NaOH to the middle of the reaction, and the employment of harsh conditions, such as high temperatures (500 °C/4 h) for calcining the product [24].

In this work, we present an innovation in the green synthesis of metallic copper nanoparticles, based on the use of cotton textile fibers (or commercial cellulose) as a stabilizer and reducing agent. This new procedure can be considered eco-friendly to the environment, utilizing soft extraction conditions, avoiding the use of toxic reducing agents like hydrazine. Moreover, in this contribution, the optimization of the process is also reported, using low-cost starting materials, where the procedure is an efficient scalable technique, with high yields of CuNPs obtained.

## 2. Materials and Methods

### 2.1. Materials and Reagents

Copper (II) sulfate pentahydrate (CuSO_4_·5H_2_O), ACS reagent ≥98%, CAS number (7758-99-8), Molecular weight 249.69 g/mol; and poly(ethylenimine) (PEI), *M*_n_ ~ 1800 by GPC, *M*_w_ ~ 2000 by Ls, 50 wt% in H_2_O were supplied in Sigma Aldrich (St. Louis, MO, USA) and cotton (Gossypium hirsutum is the most widely cultivated cotton species in the world and treated for sale) was acquired at a local store (Saltillo, México).

### 2.2. Characterization

X-ray diffraction (XRD) (Siemens, Berlin, Germany): for this test, a Siemens D-5000 diffractometer with a scanning interval in the 2θ scale from 5 to 80°, and a scan speed of 0.02%, the radiation employed was copper Kα with a wavelength of 1.54 Å; values of 25 mA and 35 kV used for intensity and voltage, respectively. The Debye–Sherrer equation was used to determine the average particle diameter through calculations at the most intense peak of the XDR diffractograms of each sample [8].

Thermogravimetric analysis (TGA) (TA instruments Inc., New Castle, Pennsylvania, PA, USA): to analyze the thermal behavior of cellulose, a DuPont Instruments 951 analyzer was utilized. Operating conditions were a heating rate of 10 °C/min, and an air atmosphere with a gas flow of 50 mL/min. The runs of the samples were carried out from 30 °C to 600 °C in an N_2_ atmosphere. Once 600 °C was reached, the N_2_ atmosphere was changed by O_2_.

Differential scanning calorimetry (DSC): this analysis was performed on a modulated TA Instrument 2920, at a heating rate of 10° C/min at a temperature range from 40 °C to 300 °C. Through a primer heating cycle, the thermal history of the samples was eliminated before obtaining the second run.

Fourier transform infrared spectroscopy (FTIR) (Perkinelmer Inc., Waltham, MA, USA): to obtain ATR spectra of selected samples of cellulose (KBr pills or cases), an FTIR Spectrometer GX, PerkinElmer was employed.

Ultraviolet-visible spectroscopy (UV-Vis) (Shimadzu Corporation, Kyoto, Japan): aqueous solutions of each sample were analyzed using a UV-visible Spectrophotometer Shimadzu MultiSpec-1501, with photodiode array and a quartz cell in absorbance mode with a tungsten lamp at a wavelength of 200–800 nm.

### 2.3. Green Synthesis of Copper Nanoparticles

In a 1 L stainless steel reactor, an aqueous solution of CuSO_4_·5H_2_O (2.5–10 g/250 mL H_2_O) was introduced, adding 1.8 mL of Poly(ethylenimine) (PEI) and 10.0 g of cotton fibers, previously washed with 100 mL of distilled water. The reaction mixture remained in agitation at 300–500 rpm for 2 h, using a mechanical shaker, setting the reaction temperature at 160–170 °C. After that time, the reactor was allowed to cool at room temperature, and then the mixture was filtered for separating the resultant solid and liquid products. The solid was washed with distilled water and dried in a vacuum stove at 80 °C for 2 h for further characterization by XRD, TGA, DSC and ATR-FTIR. The solid products obtained were identified as CCuNP2.5, CCuNP5.0 and CCuNP10.0, according to the initial addition of CuSO_4_·5H_2_O. The filtrated fluid of each reaction (aqueous filtrated solution) was analyzed by UV-Vis spectroscopy. Original cotton fibers or commercial cellulose (UT-C) were also treated at the same hydrothermic reaction conditions mentioned above, and were denoted as HT-C.

## 3. Results and Discussion

### 3.1. X-ray Diffraction

**Figure 1:**
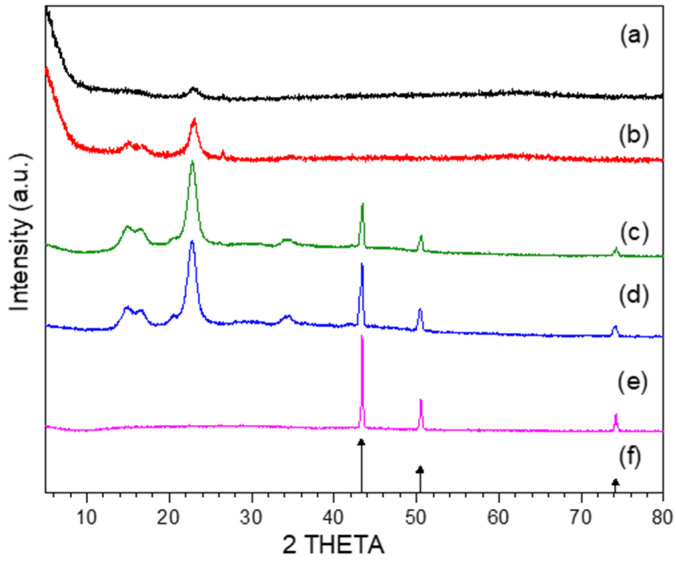
XRD patterns of nanoparticles obtained at different contents of precursor salt and cotton: (a) cotton (UT-C), (b) heat-treated cotton (HT-C), (c) CCuNP2.5, (d) CCuNP5.0, (e) CCuNP10.0, and (f) standard copper XRD (JCPDS 4–836).

Figure 1 shows the X-Ray diffraction patterns belonging to the samples UT-C, HT-C and CCuNP nanocomposites. UT-C and HT-C diffractograms clearly present the expected broad peaks of the cellulose at 14.5°, 16.4° and 22.8° (Cellulose I) corresponding to the Miller indices of (1$\bar{1}$0), (110) and (200), respectively [25]. While diffractograms of CCuNP2.5 and CCuNP5.0 nanocomposites show refractions of the cellulose and three peaks corresponding to metallic copper (Cu°) at 43.3°, 50.4° and 74.1° of the 2θ scale; the CCuNP10.0 sample only presented the peaks of metallic copper [8,9].

The last observation suggests the destruction of the cellulose structure due to the reaction conditions and the high copper salt content employed during the CCuNP10.0 synthesis.

It should be noted that all diffractograms of the obtained nanocomposites present the signals of metallic copper at 43.3°, 50.4° and 74.1°, corresponding to the Miller indices (111), (200) and (220), respectively, while impurities such as CuO, Cu_2_O or Cu(OH)_2_ commonly observed in CuNPs syntheses in aqueous media by the XRD technique were not observed.

The size of copper nanoparticles in the nanocomposites was estimated from the corresponding X-ray diffraction peak employing the Debye–Scherrer’s formula [8].

D = κλ/(β cosθ) (1)

where κ is an empirical constant equal to 0.94, λ is the wavelength of the X-ray source (1.5405 Å), β is the full width at half maximum of the diffraction peak and θ is the angular position of the peak. The estimated nanoparticle diameters were 15.6, 13.7 and 35.5 nm for CCuNP2.5, CCuNP5.0, and CCuNP10.0 respectively.

The crystallinity index (CI) of the UT-C, HT-C, CCuNP2.5, CCuNP5.0 and CCuNP10.0 was calculated using the following equation [26]:

(2) $$CI_{XRD}=\frac{\Sigma_{\left(area\;of\;crystalline\;peaks\right)}}{\Sigma_{\left(area\;of\;crystalline\;and\;amorphous\;peaks\right)}}\times 100\%$$

The crystallinity indexes estimated from Equation (2) for untreated and treated cotton fibers, comparing to the corresponding crystallinity values of the obtained nanocomposites, are exhibited in Table 1.

The observed crystallinity increase suggests that heating at 160–170 °C and the chemical treatment applied to the cotton fibers had a clear effect on the cellulose structure [26,27,28,29]. These results point out that the copper nanoparticles synthesized with contents of 2.5 g and 5.0 g of the precursor salt tend to eliminate the amorphous phase of cellulose, increasing the crystalline phase. A different case occurs in the CuNP10.0 synthesis using higher concentrations (10 g) of the precursor salt, where the amorphous and crystalline phases are completely degraded at these reaction conditions. The last results suggest the complete destruction of polymeric chains of cellulose, which can be attributed to the hydrolysis effect from the reaction medium (water) forming hydronium ions (H_3_O^+1^) at the reaction conditions employed, where copper nanoparticles can participate and catalyze the fragmentation of cellulose bonds at much lower temperatures [30].

### 3.2. Thermogravimetric Analysis (TGA)

**Figure 2:**
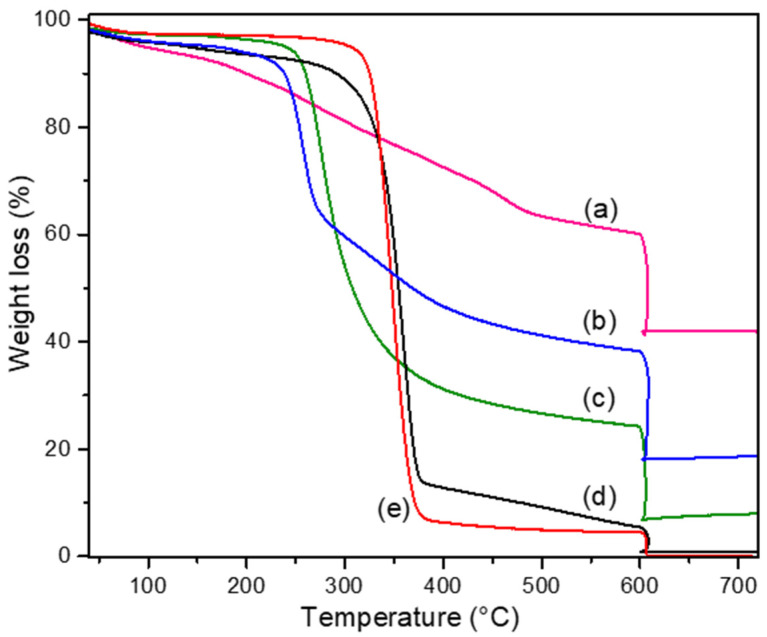
TGA curves for: (a) CCuNP10.0, (b) CCuNP5.0, (c) CCuNP2.5, (d) untreated cellulose, (e) heat-treated cellulose.

Figure 2 shows TGA thermograms of heat-treated cellulose, untreated cellulose and copper nanoparticles nanocomposites. The main decomposition stage of UT-C and HT-C is observed (Figure 2) in the range of 230–385 °C while those of CCuNP composites are localized at the range of 160–550 °C. The thermal degradation of UTC and HT-C occurs in one-step at approximately 360 °C and 348 °C, respectively [31,32]. In general, CCuNP nanocomposites thermograms show that the presence of copper favored cellulose degradation.

The degradation temperature of UT-C and HT-C samples, when the weight loss is of 5% (T_5wt%_) was of 140 °C and 305 °C, respectively (See Table 2), this suggests that hydrothermal treatment causes higher thermal stability at the start of heating. By contrast, an opposite behavior was observed in the CCuNP2.5, CCuNP5.0 and CCuNP10.0 nanocomposites, where the corresponding T_5wt%_ were lower than the HT-C sample, and the value of T_5wt%_ decreases as the copper content increases (Table 2). Therefore, it can be pointed out that hydrothermal treatment in the presence of copper sulfate salts decreases the thermal stability of the cellulose material at the start of heating in the process.

On the other hand, the maximum weight loss temperature (T_max_) decreases with hydrothermal treatment, where this decrease is more significant as copper content increases, which means that at these conditions the degradation of cellulose is favored. In the case of the nanocomposite CCuNP10.0 obtained with the highest copper content, a constant weight loss was observed in the temperature range of 150–600 °C.

The amount of copper nanocomposites residues at a temperature greater than 600 °C has been associated with the crystallinity index. S. Shi et al., by TGA, determined that cellulosic materials with a CI of 84.5% and 85.3% presented residues of 23.0% and 29.5% respectively [33]. This suggests that high crystallinity promotes the formation of thermally stable structures at high temperatures. The UT-C and HT-C samples with a CI of 39.4 and 48.0% respectively, presented relatively low residue amounts that did not exceed 1.0% (See Table 2); these results suggest that the hydrothermal treatment of cellulose (UT-C) increases the crystallinity, but the value of CI is not enough to form stable thermal structures.

The amount of copper nanocomposite residues was evidently higher compared to UT-C and HT-C (See Table 2). The value of the experimental residuals is well above those expected, if all the CuSO_4_⋅5H_2_O were transformed into Cu NP. For CCuNP 2.5 and CCuNP 5.0 nanocomposites, the high CI values could explain the aforementioned results. Meanwhile, for the CCuNP 10.0 nanocomposite, which does not contain a crystalline phase, the explanation can be attributed to the catalytic char-formation action of copper on cotton degradation products (for example lignin), where a similar explanation was reported for green lignin-based flame retardants [34]. Therefore, this kind of nanocomposite could be applied as a component in anti-flame formulations [35]. Data obtained from TGA curves and the theoretical residue percent calculated are summarized in Table 2.

**Figure 3:**
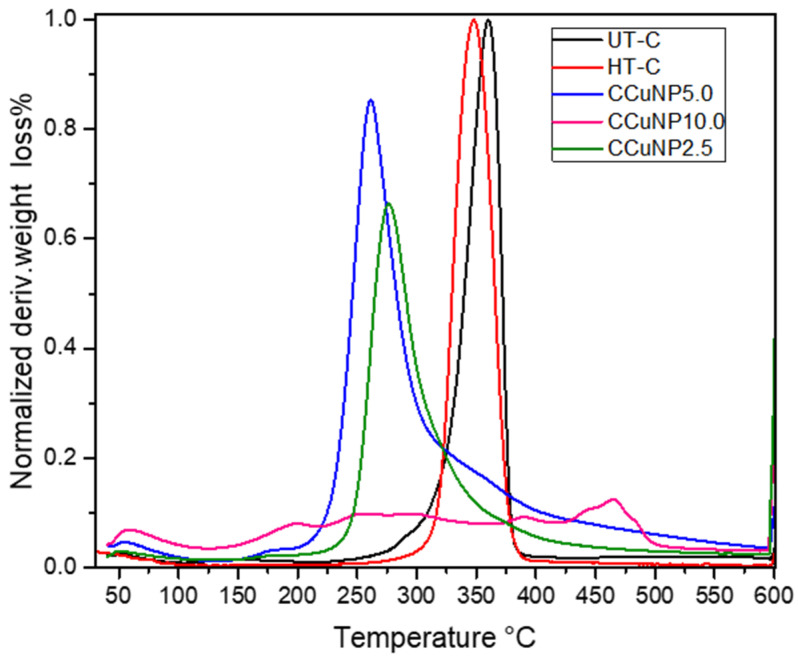
DTA curves of copper nanoparticles/cellulose (CCuNP); untreated cellulose (UT-C) and heat-treated cellulose (HT-C).

Figure 3 exhibits the DTA curves of each sample, where UT-C and HT-C showed narrow peaks, which suggest the presence of only one kind of crystal contained in these materials, while CCuNP nanocomposites displayed wide peaks, even without definition, as with the case of the CCuNP10.0 nanocomposite, suggesting the presence of heterogeneous crystals.

Making a detailed analysis of the DTA curve for CCuNP10.0 nanocomposite, we can observe four broad peaks for weight-loss at 199.2, 259.9, 390.6 and 466.2 °C. These weight losses, at 199 °C and 259.9 °C, are related to hemicellulose decomposition, as reported by Yang et al., where other research has also confirmed this observation [31,32]. On the other hand, at higher temperatures, it can be noted the peak at 390 °C that correspond to lignin decomposition, also reported by Yang’s group [32]. The T_max_ at 466.2 °C shows an important contribution to mass loss of this material, which indicates the release of aromatics, carbonyls, alkyls, CO and CO_2_, where these last two monoxide and dioxide carbons can be due to the breaking of ether bridges, linking the lignin units, or rupture of diaryl ether linkages [31]. DTA curves support and agree with the above results, and also suggest that the copper nanoparticles and the hydrothermal treatment are factors that affect the cellulose structure.

### 3.3. Differential Scanning Calorimetry (DSC)

**Figure 4:**
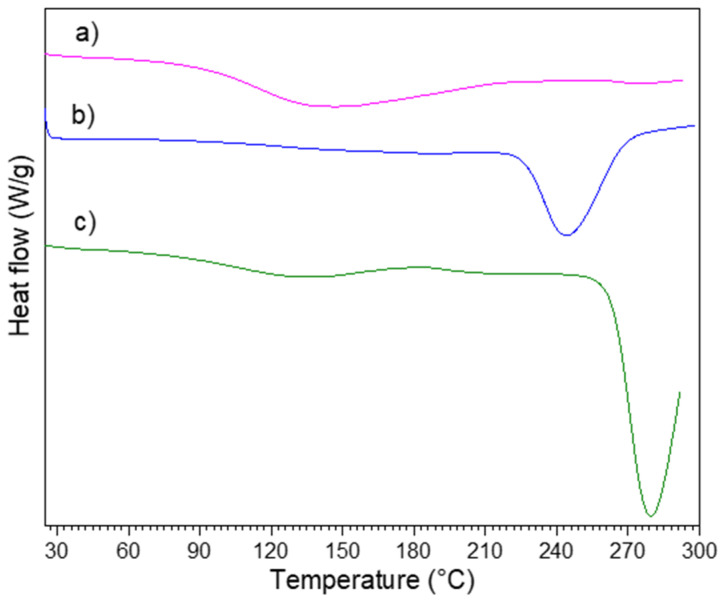
DSC curves of (a) HT-CCuNP10.0, (b) HT-CCuNP5.0 and (c) HT-CCuNP2.5.

DSC analyses of selected samples of the nanocomposites are presented in Figure 4. CCuNP2.5 and CCuNP5.0 nanocomposites showed a transition process at 279 °C and 244 °C respectively, which was attributed to hemicellulose presence [32] where, as expected, in the CCuNP10.0 nanocomposite, this thermal transition is not observed. Therefore, the last observation demonstrates that there does not exist a crystalline phase in the CCuNP10.0 structure, and most parts of the nanocomposite are amorphous. The complete degradation of the amorphous phase of the cellulose is performed in the CCuNP10.0 nanocomposite, what corroborates the obtained results by XRD.

### 3.4. Fourier Transform Infrared Spectroscopy (FTIR)

**Figure 5:**
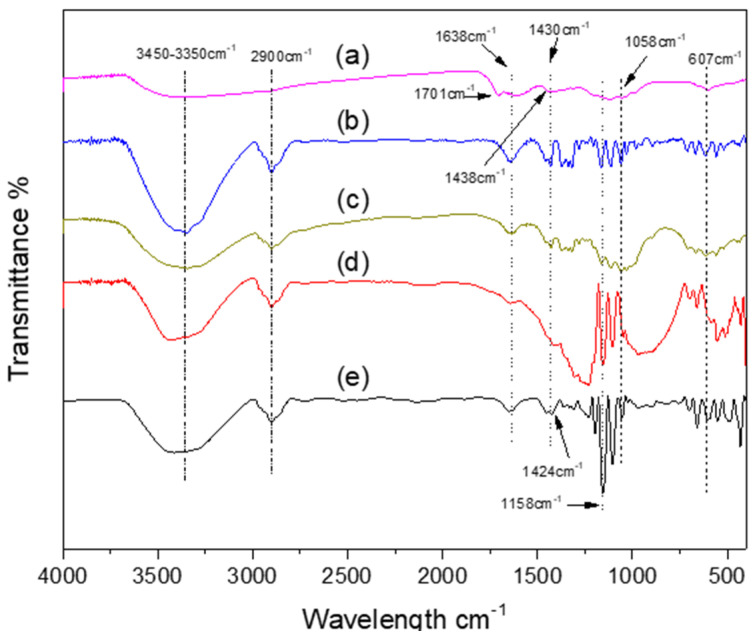
FTIR spectra of (a) CCuNP 10.0, (b) CCuNP 5.0, (c) CCuNP 2.5, (d) HT-C and (e) UT-C.

Figure 5 compares the FTIR spectra of the UT-C, HT-C, CCuNO2.5, CCuNP5.0, and CCuNP10.0 samples, showing the expected bands of the main functional groups. The absorption peaks around 3340–3400, 2900, 1638, 1427, 1059 cm^−1^, and 607 cm^−1^ are assigned to the O–H, C–H stretching, C–O stretching, crystalline absorption band, β-linkage of cellulose, C–O–C of pyrosane bonds, and to O–C–O bending vibration of this bond, respectively [26,31,34]. The presence of copper into the nanocomposites did not affect significantly the band shifts in the original FTIR spectrum of cellulose. Comparing the associated band to the crystalline region (1427 cm^−1^) of the UT-C with CCuNP2.5 and CCuNP5.0 samples, it can be observed that this band is more intense at the nanocomposites spectra, which is according to the results obtained by XRD. In addition, the characteristic broad band, corresponding to CuO around 500 cm^−1^, was not observed in any of the FTIR spectra of the nanocomposites, confirming that the CuNPs obtained by this method are stable to subsequent oxidation during the synthesis. As expected, among the nanocomposites, CCuNP10.0 samples showed a different FTIR spectrum, where the cellulose bands are not well defined, and the associated region bands to crystalline absorptions and to the β-linkage of cellulose are absent. This last observation may suggest that chemical bonds in the cellulose have been broken down, and its macromolecular structure fragmented. As can be observed in the spectrum for CCuNP10.0 sample, curve (a) in Figure 5, the band at 1638 cm^−1^ associated to C=O stretching (original cellulose) was shifted to higher frequencies at 1701 cm^−1^, which can be attributed to an oxidative process in the chemical structure of the cellulose, concerning the reaction conditions used. It is worth mentioning that FTIR spectra did not show evidence of esterification reactions in the cellulose. According to data reported in other works [31,34], the weak band located at 607 cm^−1^ belongs to lignin from cellulose, and although this vibration could be overlapped to the Cu-O band reported in the range of 502–690 cm^−1^ [36], the presence of CuO is discarded, since these types of bands are generally much stronger. Furthermore, the XRD studies confirmed that CuO is not present in any sample of the nanocomposites. After being stored for several months under atmospheric conditions, CCuNP nanocomposites demonstrated their stability, which is attributed to the protecting effect of the organic coatings preventing copper oxidation. The last results agree with the studies reported by Jardón-Maximino et al. concerning CuNPs coated with nitrogen-based ligands, finding that this type of ligand prevents the oxidation of CuNPs under atmospheric conditions over 60 months [37]. Later, the same authors found that protection of CuNPs occurs even at more drastic conditions such as in the elaboration of nanocomposites by melt processing [38].

### 3.5. UV-Vis Spectroscopy

**Figure 6:**
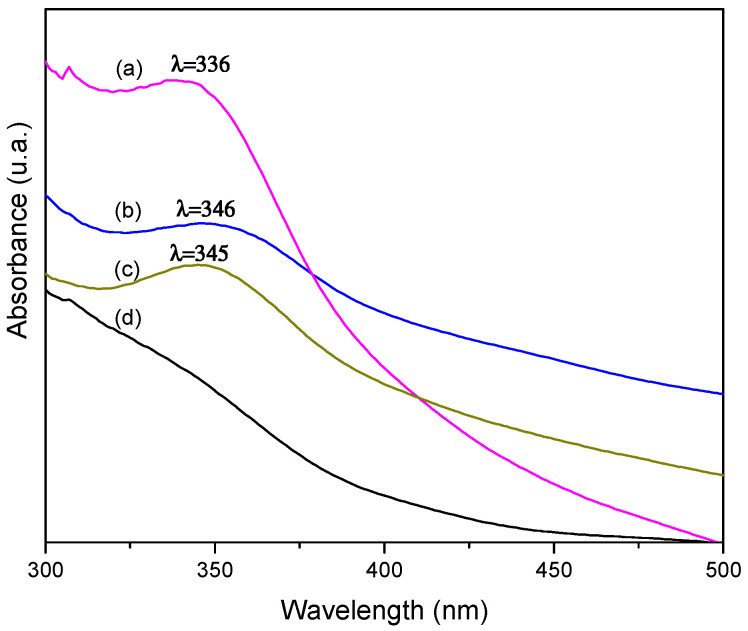
UV-Vis spectra of aqueous solutions filtrated from the original reaction fluids; (a) CCuNP10.0, (b) CCuNP5.0, (c) CCuNP2.5, and (d) CuSO_4_.

The aqueous solutions obtained from the filtration of the original reaction fluids obtained in the synthesis of the nanocomposites CCuNP were characterized by spectroscopy UV-Vis. Figure 6 shows the UV–Vis absorption spectra of the filtered aqueous solutions of each reaction, and CuSO_4_ as the precursor salt. According to data reported in other methods for the CuNPs obtainment [39], the spectra of the three nanocomposite solutions exhibit one absorption peak approximately at 340 nm, corresponding to the presence of copper oxide nanoparticles (CuO), which were solubilized in the aqueous phase, representing a simple step of purification of the CCuNPs. The CuSO_4_ UV-Vis spectrum (Figure 6d) does not show signals or bands in that wavelength range of 300–500 nm, compared to the other aqueous solutions, suggesting that copper oxidation took place.

In this regard, a new approach in the development of the present methodology can be suggested, considering that this method represents an eco-friendly and simple synthesis technique for obtaining two kinds of copper nanoparticles (Cu° and CuO), where CuO nanoparticles are obtained in aqueous solutions, and pure metallic Cu° nanoparticles stay impregnated or embedded into the cotton fibers.

## 4. Conclusions

A facile and low-cost green synthesis of copper nanoparticles from cotton textile fibers is reported in this study, which avoids the use of toxic chemical reducing agents. Copper nanoparticles sizes obtained in the range of 13–35 nm, were estimated by the XRD technique. Reaction parameters like the heating conditions and the precursor salt content were demonstrated to have a significant effect on the crystalline and amorphous phases, and degradation of the cellulose materials. The results obtained in this research confirm the development of a new simple, eco-friendly, and low-cost route for CuNPs preparation from cotton textile fibers, with potential and promising anti-flame and antimicrobial properties, which will be subsequently reported in future papers.

## Figures and Tables

**Table 1:** Crystallinity index estimated from XRD data.

Sample	UT-C	HT-C	CCuNP2.5	CCuNP5.0	CCuNP10.0
CI_XRD_ (%)	39.4	48.0	80.8	87.3	-----

**Table 2:** Data of TGA and DGA for cellulose and nanocomposites.

Sample	T_5wt%_ (°C)	T_max_ (°C)	Residue at 600 °C (%)	Theoretical Residue (%)
Untreated Cellulose (UT-C)	140	360	0.97	-
Heat-treated cellulose (HT-C)	305	348	0.04	-
CuNP 2.5	240	276	8.30	6.25
CCuNP 5.0	169	258	19.08	12.5
CCuNP10.0	96	-	41.87	25.0

## Data Availability

The data presented in this study are available on request from the corresponding author.
